# Cellular response of *Parachlorella kessleri* to a solid surface culture environment

**DOI:** 10.3389/fpls.2023.1175080

**Published:** 2023-06-05

**Authors:** Hiroki Miyauchi, Tomoharu Ishikawa, Yutaro Hirakawa, Ayumu Sudou, Katsuhiko Okada, Atsushi Hijikata, Norihiro Sato, Mikio Tsuzuki, Shoko Fujiwara

**Affiliations:** School of Life Sciences, Tokyo University of Pharmacy and Life Sciences, Hachioji, Japan

**Keywords:** attached culture, chlorella, gene expression, microalgae, photosynthesis

## Abstract

Attached culture allows high biomass productivity and is a promising biomass cultivating system because neither a huge facility area nor a large volume of culture medium are needed. This study investigates photosynthetic and transcriptomic behaviors in *Parachlorella kessleri* cells on a solid surface after their transfer from liquid culture to elucidate the physiological and gene-expression regulatory mechanisms that underlie their vigorous proliferation. The chlorophyll content shows a decrease at 12 h after the transfer; however, it has fully recovered at 24 h, suggesting temporary decreases in the amounts of light harvesting complexes. On PAM analysis, it is demonstrated that the effective quantum yield of PSII decreases at 0 h right after the transfer, followed by its recovery in the next 24 h. A similar changing pattern is observed for the photochemical quenching, with the PSII maximum quantum yield remaining at an almost unaltered level. Non-photochemical quenching was increased at both 0 h and 12 h after the transfer. These observations suggest that electron transfer downstream of PSII but not PSII itself is only temporarily damaged in solid-surface cells just after the transfer, with light energy in excess being dissipated as heat for PSII protection. It thus seems that the photosynthetic machinery acclimates to high-light and/or dehydration stresses through its temporal size-down and functional regulation that start right after the transfer. Meanwhile, transcriptomic analysis by RNA-Seq demonstrates temporary upregulation at 12 h after the transfer as to the expression levels of many genes for photosynthesis, amino acid synthesis, general stress response, and ribosomal subunit proteins. These findings suggest that cells transferred to a solid surface become stressed immediately after transfer but can recover their high photosynthetic activity through adaptation of photosynthetic machinery and metabolic flow as well as induction of general stress response mechanisms within 24 h.

## Introduction

Microalgae convert CO_2_ into organic compounds, including proteins, carbohydrates, and lipids, through photosynthetic reactions and store starch and/or neutral lipids under various stress conditions (Hamed, 2016; Okada et al., 2020). Since microalgae generally show higher photosynthetic productivity than land plants, they are expected to be promising producers of high-value-added compounds. The physiological characterization of microalgae during cultivation is thus important for the development of more efficient systems for their mass cultivation and high-value-added compound production. Numerous studies have thus far been performed for the development of new culturing systems and, in many cases, for that of suspended culture, with a focus mainly on the growth rate, photosynthesis, nutrient utilization efficiency, and metabolic flow of photosynthetic products.

Most microalgae in nature inhabit not only aquatic environments but also soil. We previously proposed that solid-surface culturing of *Parachlorella kessleri* was one of the most promising systems for algal culturing in view of its potential for high biomass production per unit area (Miyauchi et al., 2020). However, *P. kessleri* cells in solid-surface culture, relative to those in liquid culture, showed that the rate of photosynthetic CO_2_ fixation was lower in ordinary air (400 µmol CO_2_ mol air^−1^) than in air enriched with CO_2_ (2,000 µmol CO_2_ mol air^−1^), which suggested a limitation of photosynthesis in solid-surface cells by CO_2_, which slowly dissolves and diffuses (Hajer and Bayless, 2020). Additionally, light, nutrient, and water availability and the extent of dehydration on the solid surface were different from those in liquid, which might have affected the photosynthetic rate (Blanken et al., 2014; Li et al., 2015; Lai et al., 2020).

In algal cells, upon exposure to environmental stresses that damage metabolic processes, e.g., the combinatory stress of high light and low CO_2_, the cellular level of chemical energy becomes too high, causing over-reduction of the photosynthetic electron transport chain, which induces the production of reactive oxygen species (ROS) or oxidative stress that threatens cell survival (Vass et al., 2007). Photosynthetic organisms have developed protective mechanisms against such over-reduction in the photosynthetic electron transport chain, such as non-photochemical quenching (NPQ) and alternative electron flow, and ones against ROS, including the synthesis of antioxidants and the induction of antioxidative enzymes (González et al., 2021). Meanwhile, microalgae acclimate to salt stress, which causes ionic imbalance besides oxidative stress, through the release of harmful ions by the transport system and the accumulation of compatible solutes (Noctor and Foyer, 1998; Shetty et al., 2019; Kumari and Rathore, 2020).

Our solid-surface culturing system previously enabled *P. kessleri* cells to achieve a high growth rate; however, the metabolic and gene-expression regulatory mechanisms underlying the physiological properties of the cells have remained elusive. This study investigated the regulation of photosynthesis and the response of gene expression through transcriptomic analysis of RNA-Seq in *P. kesssleri* cells on a solid surface after their shift from liquid culture to understand the mechanism underlying their acclimation to solid-surface culturing conditions.

## Materials and methods

### Algal strain and growth conditions

*P. kessleri* 11h (IAM C-531 strain, the University of Tokyo; the same strain as NIES-2160, National Institute for Environmental Studies) was grown photoautotrophically in one-fifth concentration liquid Gamborg’s B5 medium (1/5 GB5) (Gamborg et al., 1968) at 30°C and 80 μmol photons m^−2^ s^−1^ under continuous illumination (fluorescent lamps, FL20S BRF; Toshiba Lighting and Technology Corporation, Japan).

For the attached culture, cells that had been first cultured with aeration with air containing CO_2_ (2,000 µmol CO_2_ mol air^−1^) for 24 h (till the log phase) and then with ordinary air (400 µmol CO_2_ mol air^−1^) for 24 h (OD730 = 0.86) were attached to a glass fiber membrane filter (GF/B Φ47 mm; Whatman, Kent, UK) at 30 mg chlorophyll (chl) m^−2^ by aspiration. Twelve membrane filters were set on a piece of non-woven fabric dampened a little with the medium in an acrylic square petri dish. The medium was infused onto the non-woven fabric in the dish (Miyauchi et al., 2020) and then circulated at 24 ml min^−1^. The solid surface was continuously irradiated with fluorescent lamps (FL20S BRF; Toshiba Lighting and Technology Corporation, Japan) at 80 µmol photons m^−2^ s^−1^. Ordinary air containing 400 µmol CO_2_ mol air^−1^ was introduced into the petri dish, with the temperature inside the device kept at 30°C.

### Measurement of the cell amount on a solid phase

Cells attached to glass fiber membrane filters were dried at 105°C for 4 h. The dry cell weight (DCW) was determined as the difference in weight between filters with and without attached cells.

For measurement of chl, a cell-attached glass fiber membrane filter was immersed in 30 ml of methanol, vortexed, and then incubated in a cool, dark place for 30 min. The methanol extract was centrifuged for 10 min, and the absorbance of the supernatant at 650 nm and 665 nm was measured. The chl amount and chl *a*/*b* ratio were calculated according to the method of Porra et al. (1989).

### Microscopic observation of the surface and cross sections of the solid phase

*Parachlorella* cells on a solid surface were observed under a scanning electron microscope (SEM) (JCM-5700; JEOL Ltd., Tokyo, Japan). For observation of cross sections, fragments of the solid phase were embedded in Tissue-Tek O.C.T. compound (Sakura Finetek USA Inc., Torrance, California, USA), and then frozen in liquid nitrogen. The solid phase was cut to a thickness of 20 μm with a cryostat microtome (HM500; Microm International, Walldorf, Germany), and the cross sections were immediately observed under a fluorescence microscope (BZ-X700; Keyence, Japan).

### Determination of cell size

Cells on a filter were suspended in the medium; about 20 μl of the suspension was placed onto a slide glass plate, and then the diameters of individual cells were measured with an automated cell counter (Cellometer ×2; Nexcelom Bioscience, USA).

### Analysis of chl fluorescence and gas exchange

The chl fluorescence measurement in liquid was carried out using a liquid phase PAM fluorometer equipped with an optical unit, a photodiode detector unit, and a PAM data acquisition system (AquaPen AP 110/C, Photon Systems Instruments, Drásov, Czech Republic), while that in solid phase was performed with a solid phase PAM fluorometer for plant leaves (Junior-PAM, Heinz Walz, Effeltrich, Germany). For the attached culture, the aspiration onto the filter was conducted under room light conditions as quickly as possible (in a couple of minutes), and then steady-state fluorescence parameters were measured for dark-adapted cells under conditions in which saturated light was pulsed (for the liquid phase, 1,200 µmol photons m^−2^ s^−1^, 800 ms; for the solid phase, 7,000 µmol photons m^−2^ s^−1^, 400 ms) every 10 s in the dark or with actinic light irradiation (80 µmol photon m^−2^ s^−1^). Light intensity-dependent fluorescence parameters were measured by irradiation with a saturated light pulse (for the liquid phase, 1,200 µmol photons m^−2^ s^−1^, 800 ms; for the solid phase, 7,000 µmol photons m^−2^ s^−1^, 400 ms) every 60 s for 360 s while increasing the intensity of the actinic light stepwise (0–500 µmol photon m^−2^ s^−1^) (**Figure S1**). *F*_v_/*F*_m_, NPQ, qP, and ΦII under each condition were calculated according to the method of Consalvey et al. (2005).

### Preparation of total RNA

Cells that had been cultured in the liquid medium (n = 3) or transferred to the solid surface and kept for 12 h or 24 h (n = 3 for each) were suspended in resuspension buffer (0.3 M sucrose, 10 mM CH_3_COONa, 20 mM EDTA, pH 7.2), and then centrifuged at 2,200*g* for 10 min at 4°C. The pellets were then immediately frozen in liquid nitrogen. Total RNA was extracted using a Sepasol^®^-RNA I Super G kit (Nacalai Tesque, Kyoto, Japan).

### Gene expression profiling with RNA-Seq

An mRNA sequencing library was constructed using an Ion Total RNA-Seq Kit v2 (Life Technologies, Carlsbad, USA), and the library was sequenced using an Ion PGM sequencer (Life Technologies, Carlsbad, USA). The quality of the sequenced reads was checked with the FastQC tool (Andrews, 2010), followed by the removal of low-quality bases (–nextseq-trim, Q <20), such as adapter sequences with Cutadapt (v 1.18), using Trimgalore (v 0.6.7) (Krueger, 2012). To quantify each transcript of *Parachlorella* samples, the sequenced reads were pseudo-mapped with Salmon (v 0.14.1) (Patro et al., 2017) using an index file created from the annotation of *Parachlorella* transcripts (Ota et al., 2016). Differentially expressed genes were identified using the integrated Differential Expression and Pathway (iDEP) analysis tool (v 0.96) using default parameters: false discovery rate (FDR) cutoff, 0.1, and minimum fold change, 2. For GO enrichment and pathway analysis, the Database for Annotation, Visualization, and Integrated Discovery (DAVID, version 6.7) (Huang et al., 2009) was employed. Because the gene annotation data for *Parachlorella* was not available in the DAVID tool, the BLAST program (Altschul et al., 1997) was used to identify orthologous genes of *Chlorella variabilis*, which is the closest organism to *Parachlorella* and for which genome annotation data was available in the tool. If a gene of *C. variabilis* hits a transcript of *Parachlorella* with a certain significance (E-value <10^−4^), the transcript was a product of the orthologous gene of *C. variabilis*.

## Results and discussion

### Cell growth and changes in photosystem II fluorescence parameters in a solid-surface culture

**Figure 1:**
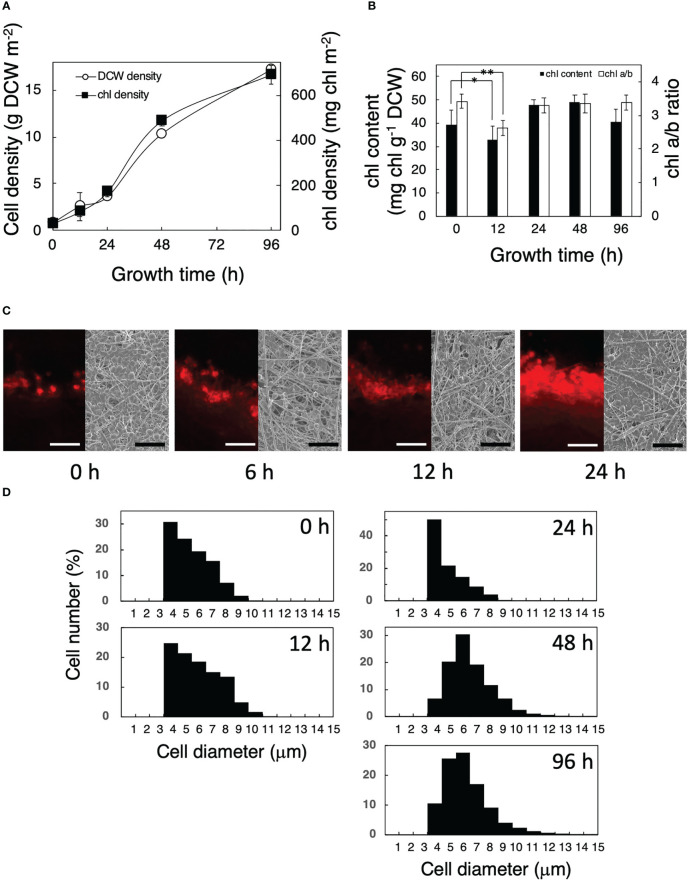
Growth of *Parachlorella* cells transferred from liquid to a solid surface. **(A)** Time courses of the dry cell weight (DCW) (○) and chl density (▪) of the cultures. **(B)** Time courses of the chl content per DCW and chl *a*/*b* ratio of the cultures. The bars represent means ± standard deviation (n = 3) (Student’s t-test, *p <0.05, **p <0.01). **(C)** Observation of a glass fiber filter on which *Parachlorella* cells had been cultivated. Left: Fluorescent micrographs of cross sections of the filter. Scale bars = 100 μm. Right: SEM images of the surfaces of the filter. Scale bar = 50 μm. **(D)** Cellular diameter of *Parachlorella* that had been cultivated on a glass fiber filter (n = 9,851, 431, 605, 6,579, and 13,788 cells in 0 h, 12 h, 24 h, 48 h, and 96 h, respectively). The diameters of algal cells cultured in liquid and immediately after being transferred to a glass fiber filter were 5.3 ± 1.4 μm (n = 4,729) and 5.4 ± 1.4 μm (n = 643), respectively, showing little effect from the aspiration. The filters on which cells had been attached at 30 mg chl m^−2^ were incubated for appropriate times.

**Figure 2:**
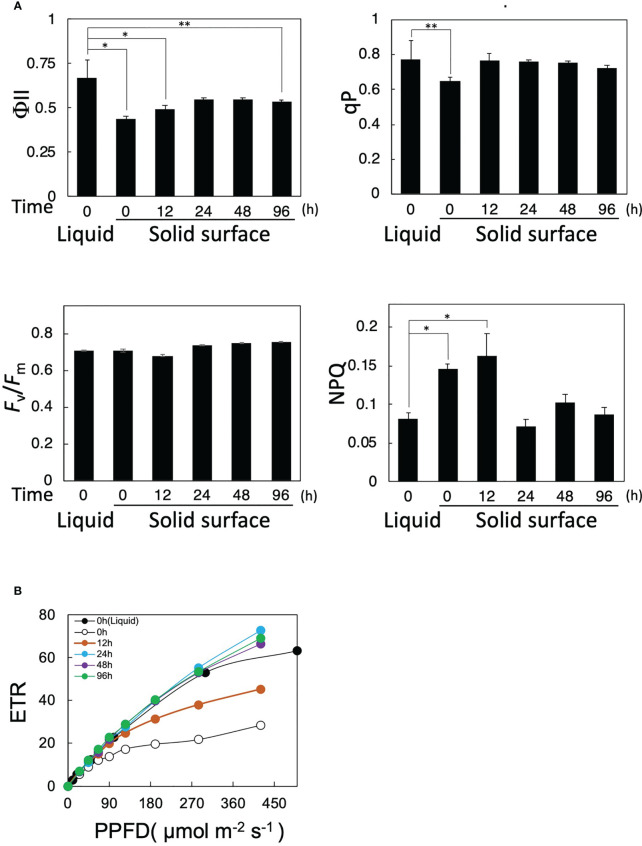
Effects of solid-surface cultivation on photosynthesis in *Parachlorella*. **(A)** Time courses of PAM fluorescence parameters (*F*_v_/*F*_m_, qP, NPQ, and ΦII) of cells transferred from liquid to the solid surface. **(B)** Light intensity-dependence curves of the ETR of cells transferred from liquid to the solid surface. The bars represent means ± standard deviation (n = 3) (Student’s t-test, *p <0.05, **p <0.01).

Growth and photosynthetic properties were investigated in *P. kessleri* cells after their transfer from liquid to solid-surface culturing conditions (**Figures 1**, **2**). Cells could grow on the solid surface (**Figure 1A**) as fast as in liquid culture (see Figure 4C of Tsuzuki et al., 2019). The chl content decreased from 39 to 33 mg chl g^−1^ DCW in the first 12 h after the transfer to the solid surface, concomitantly with a decrease in the chl a/b ratio from 3.4 to 2.6 (**Figure 1B**). These results suggested that the transfer caused the contents of the photosystem complexes to increase more slowly than those of the rest of the cellular components, and as a result, the ratio of the light-harvesting complexes to the reaction center relatively increased. Both the chl content and chl a/b ratio recovered to their initial levels in the next 12 h (**Figure 1B**). The chl content on the solid surface, which would represent the cell density, increased from 30 to 170 mg chl m^−2^ in the first 24 h, which corresponded to an increase in the number of stacked cell layers from one to several (**Figure 1C**). Since cells transferred to the solid surface at a low density would have absorbed more light energy than those in a liquid preculture, the chl decrease in the first 12 h seemed to represent cellular acclimation to high-light stress in addition to the transfer process stress itself; the chl decrease would help prevent the photosystems from absorbing excessive light energy and thus repress the generation of toxic reactive oxygen species. The later recovery in chl would indicate that the increase in the stacking number of cell layers helped relieve cells from the high-light and transfer stresses. Meanwhile, it was of note that cell volume had increased by ca. 2-fold at 48 h relative to 0–24 h (**Figure 1D**). Light intensity was taken as being the same for both liquid and solid cultures. Actually, however, the OD_730_ of a liquid culture was 0.86 when it was sampled, and the diameter of the culture tube was 2.8 cm; this means that self-shading was occurring in the liquid culture [up to (1–10^−0.86 ∗ 2.8^) ∗ 100 = 99.6 (%) when cells were farthest away from the light side], and the average light intensity in the liquid culture was much lower than the cells experienced on the glass fibers, especially until around 12 h. However, the situation seemed to change 48 h after the transfer, since several layers of cells growing on top of each other were apparent.

We then examined the effects of cell transfer from liquid to solid-surface culturing conditions on the functional changes of photosynthesis by the PAM fluorescence method. ΦII immediately decreased from 0.65 to 0.42 just after the transfer at 0 h and then gradually increased to 0.55 by 24 h, with the recovered level remaining almost steady until 96 h (**Figure 2A**). qP decreased from 0.78 to 0.62 at 0 h just after the transfer and then recovered to the level before the transfer within 12 h, whereas Fv/Fm was maintained at 0.68–0.73 throughout the solid-surface culturing for 96 h. These results implied that photosynthesis was damaged in some photosynthetic processes downstream of PSII but not in PSII right after the transfer, and that the damage was repaired within 12 h. Intriguingly, the light-intensity-dependent curve of electron transfer rate (ETR) showed that photosynthesis in solid-surface cultured cells at 0 h, as compared with liquid-cultured ones, became more sensitive to high-intensity light, showing ETR saturation at lower light intensities (**Figure 2B**). With time, however, photosynthesis began not to show the high-light-induced impairment, indicating almost the same light-intensity dependency of ETR at 24 h as that before the shift (**Figure 2B**). These observations indicated that photosynthesis became more sensitive to high light in *P. kessleri* cells right after the transfer from liquid to solid-surface culturing conditions and that it took at least 24 h for cells to recover their robustness because of the high-light stress. Meanwhile, NPQ was temporarily increased by ca. 2-fold in solid-surface cells at 0 h and 12 h, followed by a decrease at 24 h to the initial level before the transfer, with the decreased level remaining unaltered until 96 h (**Figure 2A**). No deleterious effects of the cell transfer on Fv/Fm or PSII activity would therefore be achieved through the protection of PSII by the dissipation of excessive light energy as heat. Collectively, the cell transfer to the solid surface causes some damage to a photosynthetic process downstream of PSII in *P. kessleri*, the damage being more seriously observed with illumination of light at higher intensities. Since the damage is detected immediately after the transfer, cells are affected by the transfer process itself just after the transfer and then influenced by both the transfer and high-light stresses. Nevertheless, the cells begin to recover from the photosynthetic impairment as early as 12 h, simultaneously with downregulation of the cellular contents of photosystems and PSII protection through increased thermal dissipation. Consequently, the cells can complete acclimatization to the solid-surface conditions within 24 h. A future study will be necessary for the specification of the damaged photosynthetic process and the elucidation of the photosynthesis recovery mechanism.

### Gene expression analysis in solid surface cultures

#### Regulatory expression of the primary metabolic genes in *P. kessleri* after the imposition of solid-surface stress

**Figure 3:**
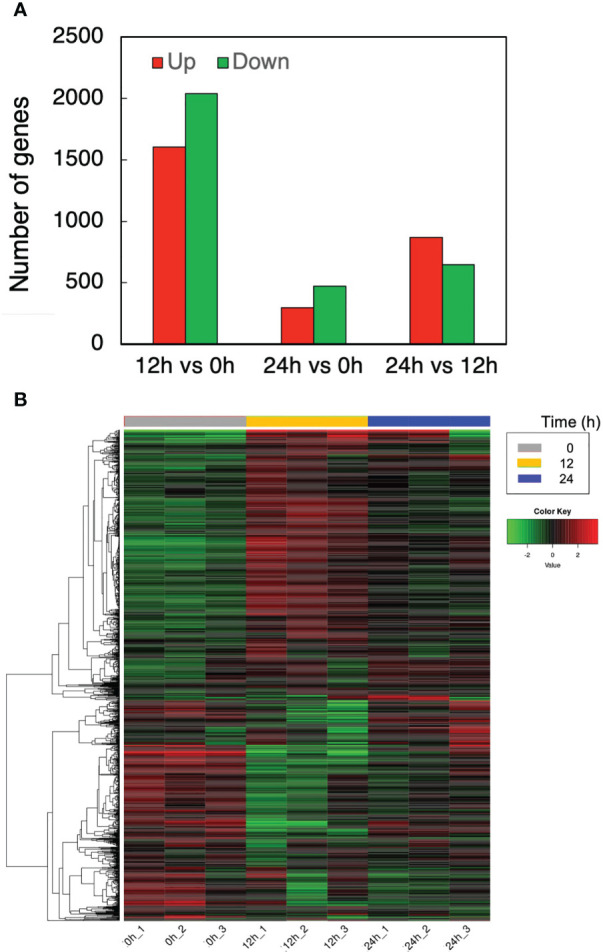
Transcriptome response to the solid surface cultivation. **(A)** The numbers of upregulated and downregulated genes. **(B)** Differentially expressed genes at different time points.

**Figure 4:**
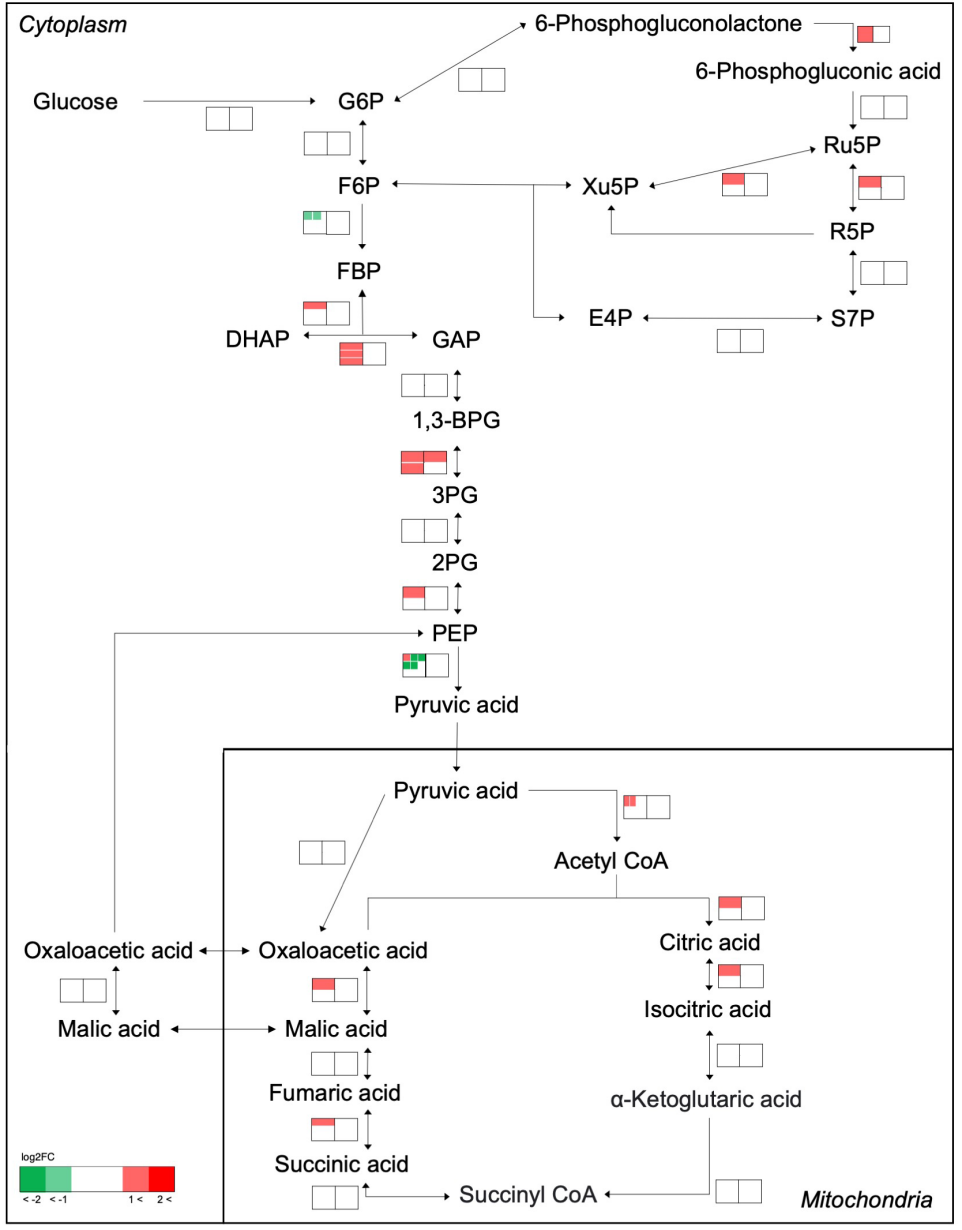
Transcriptome response of enzymes involved in the glycolysis, pentose-phosphate, and TCA cycles. The metabolic network was reconstructed based on KEGG pathway analysis. Transcriptome analysis before and after transfer to a solid surface is shown near the pathway as a heat map. The left and right heat maps show fold changes in the transcript levels at 12 h and 24 h relative to 0 h, respectively. If an enzyme is encoded by several genes, the transcript levels are indicated for each gene within the heat map and shown as separate small boxes.

**Figure 5:**
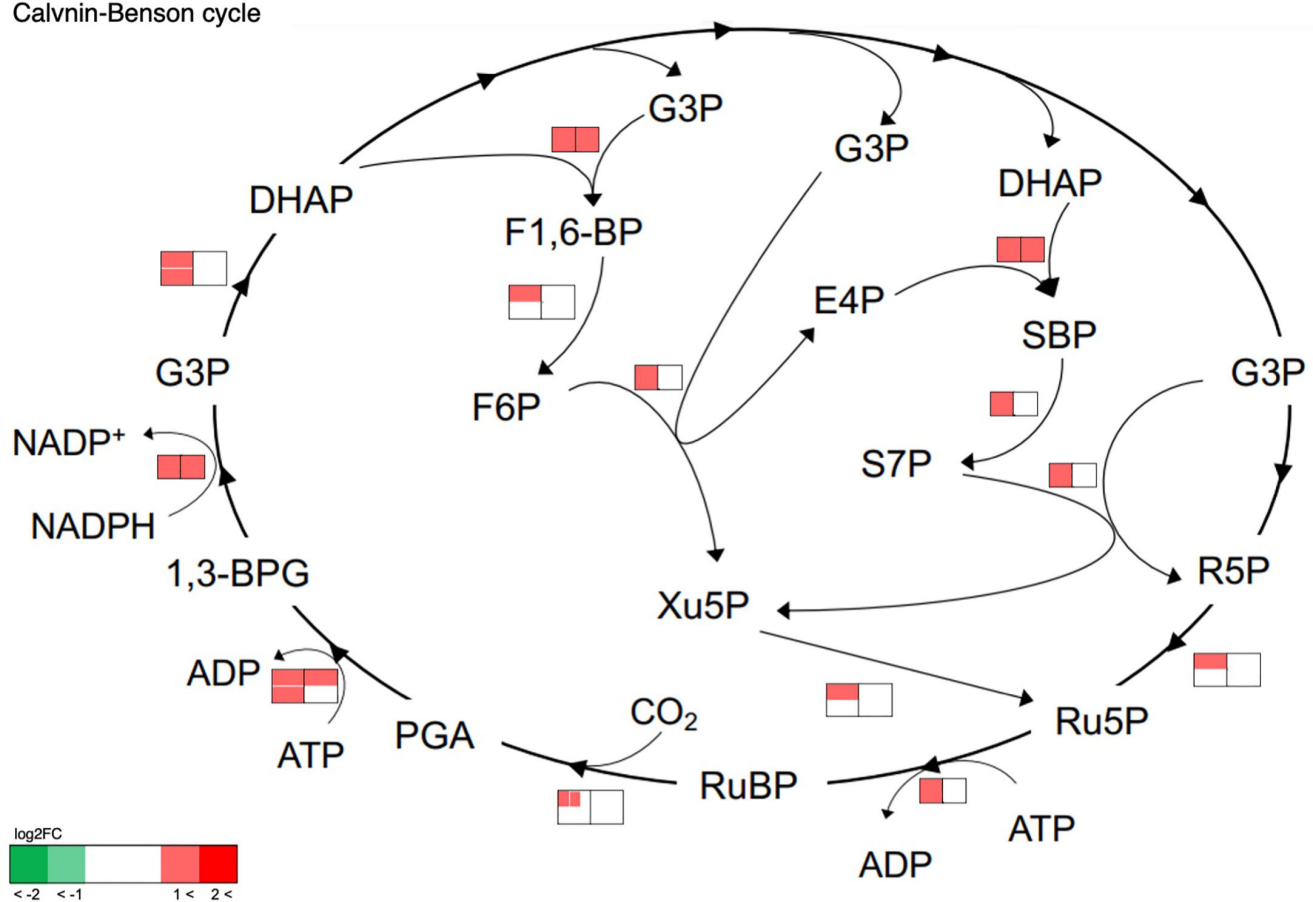
Transcriptome response of enzymes involved in the Calvin–Benson cycle. The metabolic network was reconstructed based on KEGG pathway analysis. Transcriptome analysis before and after transfer to a solid surface is shown near the pathway as a heat map. The left and right heat maps show fold changes in the transcript levels at 12 h and 24 h relative to 0 h, respectively. If an enzyme is encoded by several genes, the transcript levels are indicated for each gene within the heat map and shown as separate small boxes.

The effects of cell transfer from liquid to solid-surface conditions on the expression levels of individual genes on the genome were profiled in *P. kessleri* through transcriptomic analysis by means of RNA-Seq (13,057 genes) (Ota et al., 2016). We observed that 3,642 and 768 genes showed significantly altered expression levels (≥2 in fold change, <0.1 in FDR) in solid-surface cultured cells at 12 h and 24 h, respectively, relative to liquid-cultured cells at 0 h (**Figure 3A**). On heat map analysis, the top 3,600 variable genes were found to belong to two large clusters that showed increases and decreases, respectively, in expression levels at 12 h (**Figure 3B**). Among all the genes in *P. kessleri*, 1,642 were linked to gene IDs in *C. variabilis* through a BLAST search, with 345 and 175 genes being included in the upregulated and downregulated genes, respectively, at 12 h. These differentially expressed ID-attached genes in *P. kessleri* were then subjected to GO enrichment and KEGG pathway analyses with DAVID. GO enrichment analysis (**Table 1**) demonstrated the following GO terms for upregulated genes at 12 h relative to 0 h before the shift: translation, protein folding, ribosome biogenesis, and tRNA aminoacylation for the process of protein translation (**Table S1**); cellular amino acid, branched-chain amino acid, leucine, and arginine biosynthetic processes for the process of amino acid biosynthesis; and the tricarboxylic acid cycle and glycolytic process that are required for the synthesis of metabolic intermediates in the amino acid biosynthesis process. Consistent with the results of GO enrichment analysis, KEGG pathway analysis (**Table 2**) indicated upregulation at 12 h of a series of genes, which are involved in glycolysis (**Figure 4**, **Table S2**), pentose-phosphate (**Figure 4**, **Table S3**), TCA cycles (**Figure 4**, **Table S4**), the amino acid biosynthetic pathway (**Table S5**), and the Calvin–Benson pathway (**Figure 5**, **Table S6**). Also, we would like to characterize the adaptive process of photosynthesis under solid surface culture conditions in more detail by measuring metabolites in the near future.

**Table 1:** Biological process terms enriched by upregulated and downregulated genes (12 h *vs* 0 h).

GO ID	Term	Gene number		
		Upregulated	Downregulated	Analyzed
Upregulated				
GO:0006412	Translation	21	0	54
GO:0006457	Protein folding	11	0	17
GO:0042254	Ribosome biogenesis	6	0	10
GO:0006418	tRNA aminoacylation for protein translation	5	0	5
GO:0006189	*de novo* IMP biosynthetic process	5	0	5
GO:0008652	Cellular amino acid biosynthetic process	5	0	6
GO:0006782	Protoporphyrinogen IX biosynthetic process	5	0	6
GO:0006099	Tricarboxylic acid cycle	5	0	13
GO:0009082	Branched-chain amino acid biosynthetic process	4	0	5
GO:0006730	One-carbon metabolic process	4	0	5
GO:0006096	Glycolytic process	4	0	11
GO:0009098	Leucine biosynthetic process	3	0	3
GO:0001732	Formation of cytoplasmic translation initiation complex	3	0	3
GO:0006526	Arginine biosynthetic process	3	0	4
Downregulated				
GO:0006270	DNA replication initiation	0	5	5
GO:0007018	Microtubule-based movement	1	5	10
GO:0006260	DNA replication	0	5	13
GO:0009308	Amine metabolic process	0	2	2
GO:0051276	Chromosome organization	0	2	2
GO:0030030	Cell projection organization	0	2	2
GO:0051321	Meiotic cell cycle	0	2	2
GO:1902600	Hydrogen ion transmembrane transport	0	2	3

**Table 2:** KEGG pathway terms enriched by upregulated and downregulated genes (12 h *vs* 0 h).

KEGG pathway ID	Term	Gene number		
		Upregulated	Downregulated	Analyzed
Upregulated				
cvr01110	Biosynthesis of secondary metabolites	65	8	183
cvr01230	Biosynthesis of amino acids	31	0	59
cvr01200	Carbon metabolism	22	2	68
cvr03010	Ribosome	21	0	54
cvr00970	Aminoacyl-tRNA biosynthesis	18	0	26
cvr01240	Biosynthesis of cofactors	18	0	41
cvr00710	Carbon fixation in photosynthetic organisms	12	1	19
cvr03008	Ribosome biogenesis in eukaryotes	12	0	20
cvr01210	2-Oxocarboxylic acid metabolism	11	0	16
cvr00860	Porphyrin metabolism	9	0	16
cvr00400	Phenylalanine, tyrosine, and tryptophan biosynthesis	7	0	9
cvr00220	Arginine biosynthesis	7	1	11
cvr00250	Alanine, aspartate, and glutamate metabolism	6	0	15
cvr00290	Valine, leucine, and isoleucine biosynthesis	5	0	7
Downregulated				
cvr03030	DNA replication	0	8	13
cvr02010	ABC transporters	2	4	12
cvr00910	Nitrogen metabolism	1	3	6
cvr03430	Mismatch repair	0	3	8
cvr03440	Homologous recombination	0	3	7

**Figure 6:**
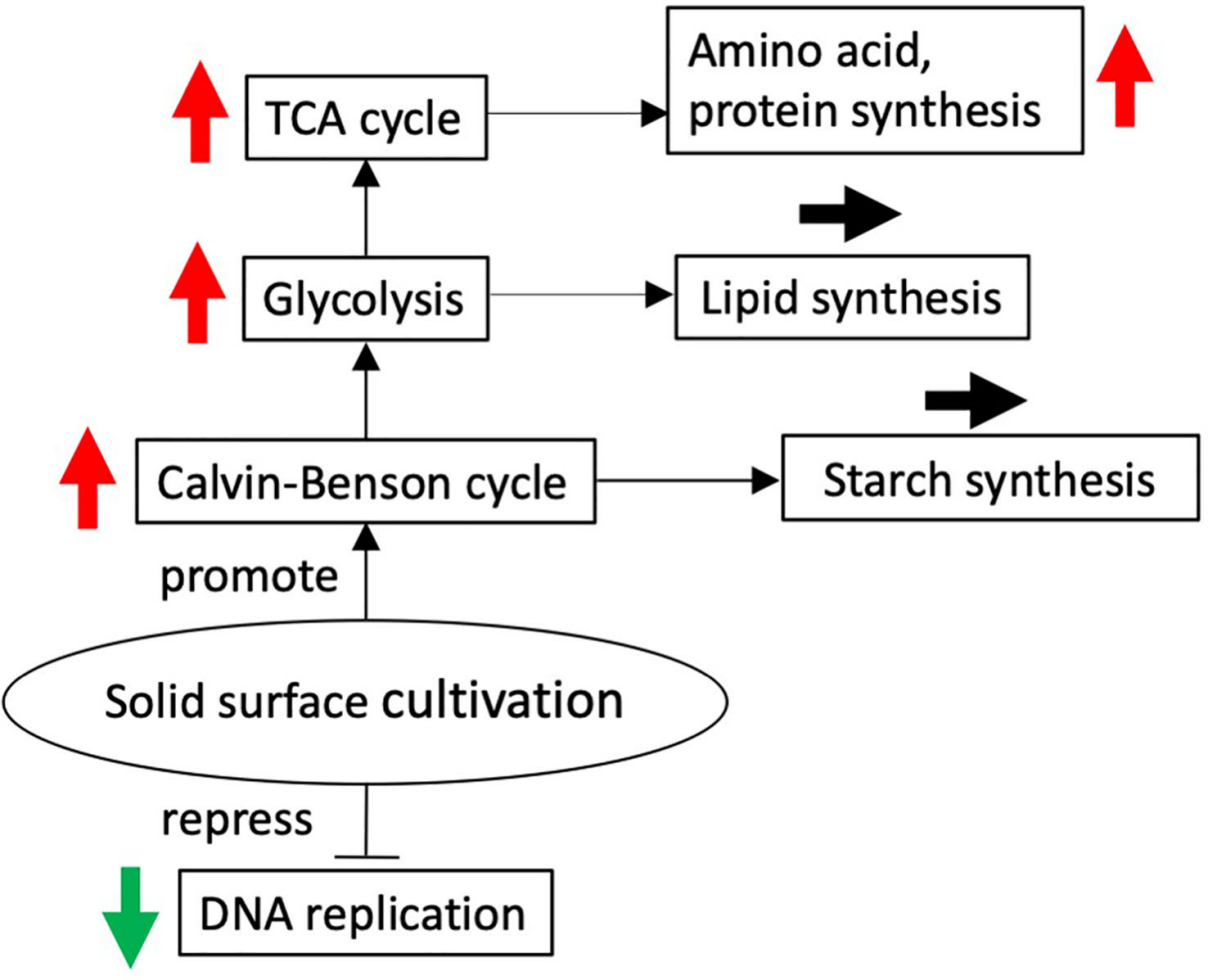
Solid surface cultivation-regulated schematic diagram. Red and green arrows show up and downregulation of differentially expressed genes at 12 h after the transfer to the solid surface compared with before the transfer, respectively.

Overall, it seems that cell transfer to solid-surface conditions leads to activation of the central metabolism for the synthesis of amino acids or building blocks for protein synthesis, in line with reinforcement of the ability of the translation system (**Figure 6**). It is, however, of note that the expression levels had become lower at 24 h for almost all the above genes, with the decreased levels reaching the initial levels in many cases (**Figures 4**, **5**, **Tables S1–S6**). The transient activation of the central metabolism, in collaboration with that of the translation system (**Table S1**), would facilitate the synthesis of proteins or enzymes that are required for the acclimation of cells just after the transfer to solid-surface conditions. The metabolic activation could be related to the production of extracellular polymeric substances (EPSs), including proteins in addition to carbohydrates, although it is not clear from our data whether the synthesis of the main carbohydrate constituents, rhamnose, xylose, mannose, and galactose, is upregulated or not (Ciempiel et al., 2022). Meanwhile, GO terms that represented downregulated genes included DNA replication and its initiation and microtubule-based movement (**Table 1**). The increase in cell size in *P. kessleri* upon the transfer of cells to solid-surface conditions might be caused by repression of DNA replication and, inevitably, cell division.

#### Regulatory expression of the genes involved in photosynthetic process on the thylakoid membrane

The physiological data presented above (**Figures 1**, **2**) indicated that the transfer of cells from liquid to solid-surface conditions affected photosynthesis by the cells; they became more sensitive to high light but then acclimated to high light within 24 h. Thus, the expression of genes involved in the photosynthetic process on the thylakoid membrane (this section, **Table 3**) and the high-light stress response (next section **Table 4**) were investigated. Generally, the mRNA levels of genes related to light-harvesting complexes and photosystem complex proteins were more upregulated than downregulated (among 39 genes for the light-harvesting complexes and photosystem complexes, only one was significantly downregulated while eight were significantly upregulated) (**Table 3**). On the other hand, the mRNA levels of the cytochrome b_6/_f complex and plastocyanin, which participate in the photosynthetic electron transport system, i.e., the proteins responsible for the supply of chemical energy that drives the above anabolic pathway, were significantly elevated at 12 h (**Table 3**). Expression of genes for chloroplast-localized ferredoxin, PGR5, and F-type ATPase subunits was also upregulated (**Table 3**), suggesting that increased PSI cyclic electron transport relieves photoinhibition and thereby reduces production of reducing power, but maintains high ATP synthesis.

**Table 3:** Relative expression levels of genes involved in the photosynthetic process on the thylacoid membrane (n = 3).

Parachlorella gene ID*	Predicted function*	12 h/0 h		24 h/0 h		24 h/12 h	
		log2 fold change	padj**	log2 fold change	padj**	log2 fold change	padj**
PSII							
452_T	light-harvesting complex II chlorophyll a/b binding protein 1	0.33	0.55	1.22	0.05	0.90	0.12
1779_T	light-harvesting complex II chlorophyll a/b binding protein 1	-1.04	0.00	-0.44	0.44	0.59	0.18
506_T	light-harvesting complex II chlorophyll a/b binding protein 2	-0.02	0.98	0.34	0.70	0.35	0.57
5532_T	light-harvesting complex II chlorophyll a/b binding protein 2	0.61	0.27	1.10	0.11	0.49	0.49
5642_T	light-harvesting complex II chlorophyll a/b binding protein 2	0.34	0.50	0.74	0.26	0.40	0.52
5644_T	light-harvesting complex II chlorophyll a/b binding protein 2	0.33	0.60	1.13	0.13	0.80	0.24
7894_T	light-harvesting complex II chlorophyll a/b binding protein 2	0.57	0.25	0.91	0.16	0.35	0.60
6790_T	light-harvesting complex II chlorophyll a/b binding protein 4	0.57	0.20	0.83	0.16	0.27	0.67
1930_T	light-harvesting complex II chlorophyll a/b binding protein 5	0.83	0.06	0.98	0.09	0.15	0.84
1915_T	light-harvesting complex II chlorophyll a/b binding protein 7	1.11	0.01	0.66	0.32	-0.44	0.46
608_T	MBB1; PsbB mRNA maturation factor	-0.20	0.63	0.21	0.79	0.41	0.39
3289_T	PSBP4; lumenal PsbP-like protein	2.03	0.00	1.18	0.05	-0.85	0.12
2084_T	photosystem II PsbY protein	0.46	0.22	0.61	0.24	0.15	0.79
2085_T	photosystem II PsbY protein	0.39	0.44	0.58	0.42	0.20	0.78
7992_T	photosystem II 10kDa protein	0.62	0.13	0.94	0.07	0.32	0.57
4238_T	photosystem II 13kDa protein	1.98	0.00	1.04	0.04	-0.94	0.03
8704_T	Photosystem II 22 kDa protein	-1.50	0.23	0.92	0.69	2.42	0.08
947_T	putative photosystem II 22 kDa protein (ISS)	0.24	0.57	0.29	0.70	0.05	0.94
2533_T	photosystem II stability/assembly factor HCF136	1.20	0.00	1.18	0.00	-0.03	0.97
3636_T	photosystem II oxygen-evolving enhancer protein 1	-0.09	0.89	-0.49	0.63	-0.40	0.61
10576_T	photosystem II oxygen-evolving enhancer protein 1	0.95	0.02	0.92	0.09	-0.03	0.97
5173_T	photosystem II oxygen-evolving enhancer protein 2	1.00	0.01	0.98	0.04	-0.02	0.97
3349_T	photosystem II oxygen-evolving enhancer protein 3	0.88	0.03	0.95	0.08	0.07	0.93
Electron transport system							
8540_T	cytochrome *b_6_f* complex iron-sulfur subunit [EC:1.10.9.1]	1.20	0.00	0.94	0.02	-0.26	0.57
10216_T	cytochrome *b_6_f* complex iron-sulfur subunit [EC:1.10.9.1]	-0.29	0.65	-0.32	0.78	-0.03	0.98
4808_T	cytochrome *b_6_f* complex subunit 8	1.86	0.00	1.35	0.00	-0.51	0.24
6097_T	plastocyanin	1.17	0.00	1.30	0.00	0.13	0.84
PSI							
914_T	light-harvesting complex I chlorophyll a/b binding protein 1	0.86	0.06	1.13	0.05	0.27	0.69
4229_T	light-harvesting complex I chlorophyll a/b binding protein 2	0.52	0.33	0.84	0.24	0.32	0.66
8690_T	light-harvesting complex I chlorophyll a/b binding protein 3	0.70	0.08	0.90	0.09	0.20	0.74
4230_T	light-harvesting complex I chlorophyll a/b binding protein 4	0.65	0.14	0.79	0.20	0.14	0.85
7758_T	light-harvesting complex I chlorophyll a/b binding protein 4	0.62	0.14	0.96	0.07	0.34	0.55
915_T	light-harvesting complex I chlorophyll a/b binding protein 5	0.68	0.10	0.91	0.09	0.23	0.71
1506_T	light-harvesting complex I chlorophyll a/b binding protein 5	-0.20	0.73	0.57	0.47	0.77	0.19
1594_T	light-harvesting complex I chlorophyll a/b binding protein 5	0.59	0.15	0.83	0.13	0.24	0.69
1505_T	LHCA2; light-harvesting protein of photosystem I	-0.43	0.38	0.54	0.48	0.97	0.06
2109_T	photosystem I subunit II	0.66	0.06	0.83	0.07	0.17	0.74
417_T	photosystem I subunit III	0.73	0.07	0.93	0.07	0.21	0.73
849_T	photosystem I subunit V	0.54	0.22	0.93	0.10	0.39	0.50
4033_T	photosystem I subunit X	0.61	0.22	0.93	0.16	0.32	0.64
6368_T	photosystem I subunit XI	0.76	0.04	0.84	0.09	0.09	0.89
7877_T	photosystem I subunit IV	0.72	0.05	0.90	0.06	0.17	0.75
1456_T	photosystem I subunit PsaO	0.69	0.09	1.00	0.06	0.31	0.59
2849_T	ferredoxin	1.40	0.00	0.31	0.76	-1.10	0.03
3374_T	ferredoxin	1.72	0.00	2.07	0.00	0.35	0.61
4501_T	ferredoxin	1.00	0.02	0.82	0.17	-0.18	0.79
7710_T	ferredoxin	2.71	0.00	2.06	0.00	-0.66	0.33
7711_T	ferredoxin	1.62	0.00	1.21	0.00	-0.41	0.37
3147_T	Ferredoxin–NADP^+^ reductase [EC:1.18.1.2]	-0.24	0.53	-0.11	0.90	0.13	0.80
5387_T	Ferredoxin–NADP^+^ reductase [EC:1.18.1.2]	0.84	0.00	0.47	0.31	-0.37	0.36
PSI cyclic electrontransport							
9452_T	thylakoid membrane protein (PGR5)	1.25	0.00	1.03	0.06	-0.22	0.73
ATP synthesis							
4976_T	F-type H^+^-transporting ATPase subunit b [EC:3.6.3.14]	1.65	0.00	0.96	0.01	-0.69	0.06
1017_T	F-type H^+^-transporting ATPase subunit gamma [EC:3.6.3.14]	1.75	0.00	0.97	0.01	-0.78	0.02
4471_T	F-type H^+^-transporting ATPase subunit delta [EC:3.6.3.14]	1.96	0.00	1.14	0.00	-0.82	0.02

*Ota et al. (2016).

**Adjusted p-value.

**Table 4:** Relative expression levels of upregulated and downregulated genes involved in the stress response (n = 3).

Parachlorella gene ID*	Predicted function*	12 h/0 h		24 h/0 h		24 h/12 h	
		log2 fold change	padj**	log2 fold change	padj**	log2 fold change	padj**
SOD							
2269_t	fsd1; superoxide dismutase [Fe]	1.32	0.00	0.89	0.02	−0.43	0.28
1919_t	superoxide dismutase, Fe–Mn family [EC:1.15.1.1]	−2.11	0.09	−3.26	0.03	−1.14	0.50
2901_t	superoxide dismutase, Fe–Mn family [EC:1.15.1.1]	−3.01	0.00	−0.15	0.95	2.86	0.01
Chaperonin							
2171_t	hypothetical protein	3.34	0.00	1.32	0.00	−2.02	0.00
2203_t	CPN60A; chaperonin 60A	2.88	0.00	0.93	0.01	-1.95	0.00
10792_t	CPN60B2; chaperonin 60B2	2.55	0.00	0.76	0.09	−1.80	0.00
11644_t	chaperonin GroES	2.39	0.00	0.71	0.10	−1.68	0.00
10300_t	molecular chaperone HtpG	2.35	0.00	0.81	0.04	−1.54	0.00
8515_t	molecular chaperone HtpG	2.11	0.00	0.54	0.28	−1.57	0.00
13036_t	chaperonin GroEL	1.73	0.00	0.57	0.33	−1.16	0.00
11885_t	molecular chaperone GrpE	1.64	0.00	0.60	0.19	−1.03	0.00
7788_t	molecular chaperone DnaK	1.61	0.00	0.43	0.51	−1.18	0.00
7471_t	chaperonin GroEL	1.57	0.00	0.31	0.73	−1.26	0.01
8607_t	PFD1; prefoldin chaperone 1	1.56	0.00	0.66	0.20	-0.90	0.02
7787_t	molecular chaperone DnaK	1.56	0.00	0.34	0.53	−1.21	0.00
9423_t	chaperonin GroES	1.55	0.00	0.57	0.15	−0.98	0.00
9781_t	molecular chaperone GrpE	1.53	0.00	0.41	0.50	−1.11	0.00
6737_t	Molecular chaperone (DnaJ superfamily) (ISS)	1.16	0.01	0.45	0.61	−0.71	0.23
8968_t	molecular chaperone DnaJ	1.10	0.00	0.32	0.48	−0.78	0.01
4644_t	DnaJ homolog subfamily C member 2	1.10	0.00	0.10	0.90	−1.00	0.01
7857_t	dnj14; molecular chaperone	1.06	0.01	0.03	0.98	−1.04	0.04
5879_t	DnaJ homolog subfamily A member 2	1.09	0.00	−0.11	0.85	−1.20	0.00
HSP							
10768_t	HSP20 family protein	2.80	0.00	0.94	0.28	−1.86	0.00
10763_t	HSP20 family protein	2.48	0.00	0.62	0.54	−1.86	0.00
5674_t	HSP20 family protein	1.97	0.00	−0.44	0.59	−2.41	0.00
1000_t	HSP70B; heat shock protein 70B	1.97	0.00	0.83	0.04	−1.14	0.00
10583_t	heat shock 70kDa protein 1/8	1.84	0.00	0.25	0.61	−1.60	0.00
2144_t	heat shock 70kDa protein 4	1.77	0.00	0.49	0.41	−1.28	0.00
2145_t	heat shock 70kDa protein 4	1.59	0.00	0.38	0.56	−1.21	0.00

*Ota et al. (2016).

**Adjusted p-value.

From the aspect of the functional regulation of photosynthesis, it was of note that the gene expression levels for thioredoxin were upregulated at 12 h, whereas those for the zeaxanthin epoxidase genes were downregulated (**Tables S8, S9**). Thioredoxin is necessary for the light activation of some members of the Calvin–Benson cycle, and thus the elevation in expression levels seems to support the activation of the Calvin–Benson cycle.

The xanthophyll cycle, which is composed of violaxanthin de-epoxidase (VDR) and zeaxanthin epoxidase (ZEP), is known as a mechanism that protects plants under high light conditions against oxidative stress. VDR activation/inactivation through ∆pH across thylakoid membranes regulates the xanthophyll cycle with the use of zeaxanthin, the product of VDR, for the dissipation of excessive light energy as heat. The mRNA levels of VDR1 (4830_t) and ZEP (740_t, minor gene for ZEP) were little changed, while those of the other ZEPs (9325_t/12660_t/12661_t, major genes for ZEP) were markedly decreased at 12 h (**Tables S7, S9**). The downregulation of the ZEP gene responsible for violaxanthin synthesis might lead to an increase in the content of zeaxanthin relative to that of violaxanthin and might thereby contribute to the elevation in NPQ after 12 h under transfer and high light stresses, although photosynthetic pigment determination is required to prove this hypothesis. It thus seems that both *PGR5* upregulation and *ZEP* downregulation play roles in the protection of PSII in *P. kessleri* cells cultured on a solid surface, especially against high-light stress.

Concomitantly upregulated were the genes involved in the protoporphyrinogen IX biosynthetic process (**Table 1**) or porphyrin metabolism (**Table 2**) for the synthesis of chl and heme, which are co-factors of the photosystem complexes and cytochromes. It is not clear why the chl content decreased at 12 h despite the upregulation of genes related to chl synthesis. In *Chlorella sorkiniana*, mixotrophic growth induced upregulation of chl synthase, but the chl content was reduced due to the decrease in the amounts of chl-binding proteins (Cecchin et al., 2018). Similarly, in this study, the accumulation of some photosynthetic proteins might be repressed by high light stress, and this could be related to the decrease in the chl content, although quantification of photosynthetic proteins is required for further discussion.

#### Regulatory expression of stress-response related genes

Upregulation of stress-response-related genes, including those of superoxide dismutase (SOD), HSP70B, and chaperonins, was another aspect of regulatory gene expression in *P. kessleri* cells at 12 h after the transfer to solid-surface conditions (**Table 4**; **Tables S7, S8**). SODs are among the most important antioxidant defense factors in photosynthetic organisms (Alscher et al., 2002). The mRNA level of a major Fe SOD (FeSOD) (2269_t), which is generally known to be localized in chloroplasts (Page et al., 2012), was increased to 2^1.3^-fold at 12 h, while those of major Mn SODs (MnSODs), probably including mitochondrial SODs (Page et al., 2012), decreased markedly (1919_t) or moderately (1964_t) (**Table 4**; **Tables S7–S9**). Interestingly, a homolog (2171_t) of chloroplast-localized co-chaperonin CHAPERONIN 20, which is a mediator of FeSOD activation through direct interaction in *Arabidopsis* (Kuo et al., 2013), was drastically upregulated at 12 h (**Table 4**; **Tables S7, S8**). Collectively, it was supposed that FeSOD in chloroplasts, but not MnSOD in mitochondria, is upregulated in gene expression at the transcript level, is activated post-translationally by CHAPERONIN 20, and thereby functions against high-light stress.

It is generally accepted that HSP70 is involved in the refolding of proteins denatured under heat-stress conditions; however, *HSP70* genes increased in expression level transiently with a peak at around 10 min–6 h upon exposure not only to heat but also to high light, salt, osmotic, or desiccation stress (Sato et al., 2007; Liu et al., 2010; Rupprecht et al., 2010; Tsuzuki et al., 2019). This study showed that the expression level of *HSP70B* increased within 12 h after the transfer of cells from liquid to the membrane surface (**Table 4**; **Tables S7, S8**), suggesting the transfer and/or the high-light stress induced the expression. To examine how HSP70B contributes to the stress response, a future study is necessary, including a more detailed time-course analysis.

In this context, it should be mentioned that upregulated amino acid synthesis genes include the pyrroline-5-carboxylate reductase one for the synthesis of Pro (**Table S5**), which, along with sucrose, trehalose, glucosylglycerate, floridoside, and Gly betaine, is known as a compatible solute for microalgal survival in hyperosmotic environments (Borowitzka et al., 2016; You et al., 2019). A previous report suggested that *P. kessleri* utilized Pro as a compatible solute, consistent with this study (You et al., 2019): *P. kessleri* cells on the solid surface might accumulate Pro to mitigate the extent of the hyperosmotic stress caused by the transfer process. Meanwhile, *C. vulgaris*, which like *P. kessleri* belongs to the Trebouxiophyceae, seemed to accumulate putrescine, xylitol, and trehaose-6-phosphate under desiccation and rehydration stresses, according to its metabolite profiling, which inferred a wide variety of compatible solutes depending on the algal strain (Aigner et al., 2022).

The solid surface culture conditions in this study do not seem to cause severe desiccation stress for *P*. *kessleri* since the filter used as the solid phase contains water and even algae with little desiccation tolerance, such as *Chlamydomonas*, can grow well on the surface. Thus, the alteration of gene expression detected in this study might be induced by stimuli unrelated to desiccation stress, as in the study with the chlorophyte *Haematococcus lacustris* (*pluvialis*) (Roach et al., 2022). These findings could provide new general insights regarding the algal response to a minor environmental change, which often occurs in the natural world. Moreover, the strain of *P*. *kessleri* (formerly *Chlorella kessleri*) used in this study had been shown to be desiccation-tolerant and to accumulate triacylglycerols under air-drying conditions (Shiratake et al., 2013). Thus, it would be interesting to know the differences in transcriptional and physiological responses between desiccation-sensitive and tolerant species of the Chlorellaceae for future research.

## Data availability statement

The original contributions presented in the study are included in the article/**Supplementary Material**. Further inquiries can be directed to the corresponding author.

## Author contributions

HM, TI, SF, and MT designed the experiments. HM and TI conducted the experiments. HM, TI, YH, and AH analyzed the data. HM, SF, NS, KO, and MT wrote the manuscript. All authors contributed to the article and approved the submitted version.
