# Supplementary material for: Cellular response of Parachlorella kessleri to a solid surface culture environment
Source: Front Plant Sci. 2023 Jun 5;14:1175080. doi: 10.3389/fpls.2023.1175080 (PMC10277731; doi:10.3389/fpls.2023.1175080)
Supplement: Supplementary file 1 [file Image_1.pdf]

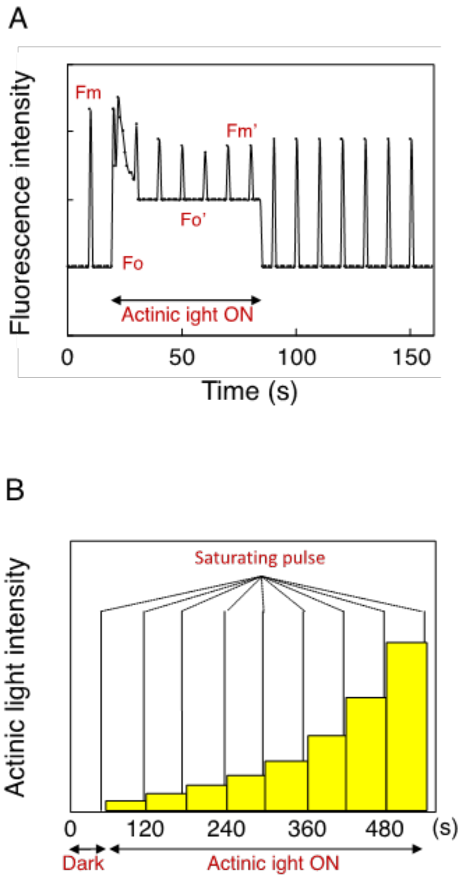

Fig. S1. Schematic representation of fluorescence quenching analysis using the saturation pulse method (pulse-amplitude modulation technique, PAM). A: Analysis under steady-state conditions. The minimum and maximum fluorescence levels (F_0 and F_m) were measured after short dark-adaptation using weak modulated measuring light and a saturating light pulse to calculate the maximum photochemical yield (F_0/F_m). After that, the sample was illuminated with a constant intensity of actinic light and a series of saturating pulses in order to reach the steady state F_0' and F_m'. B: Analysis of the light intensity-dependency. After the short dark-adaptation, the intensity of the actinic light was increased stepwise every 60s, and the fluorescence levels were measured using a saturating pulse.

.

.
